# Clinical characteristics and risk factors associated with diverse manifestations of COVID-19 in patients aged 85 years and older

**DOI:** 10.3389/fpubh.2024.1407146

**Published:** 2024-09-25

**Authors:** Zao-Xian Xu, Yi Yang, Shang-Ping Xin, Xiao-ling Shou

**Affiliations:** Department of Cardiac Rehabilitation, Zhejiang Hospital, Hangzhou, Zhejiang, China

**Keywords:** aged 85 years or older, COVID-19, disease severity, risk factors, clinical characteristics

## Abstract

**Objective:**

The goal of this study is to assess the clinical attributes exhibited by patients aged 85 years and older who present different manifestations of COVID-19, and to examine the factors influencing the classification of the disease severity.

**Method:**

This retrospective study was conducted at a single center, encompassing an analysis of clinical data obtained from patients with COVID-19 admitted to a general geriatric hospital in Hangzhou, Zhejiang, China, during the period from December 20, 2022, to February 1, 2023. The study focused on 91 eligible patients whose disease severity was compared based on the imaging findings.

**Results:**

A total of 91 patients aged 85 years and older, with a median age of 92, including 46 males, 10 exhibiting mild symptoms, 48 moderate cases, and 33 severe cases met the inclusion criteria. Notably, disease severity displayed a significant correlation with age (*p* < 0.011). All patients presented with complicated chronic underlying conditions and a history of prolonged medication use. Rheumatic immune diseases (*p* = 0.040) and endocrine medications, primarily hypoglycemic agents (*p* = 0.034), exhibited statistical significance. Additionally, markers such as lactate dehydrogenase (LDH) (*p* = 0.030), interleukin 6 (IL-6) (*p* = 0.014), and D-dimer (*p* = 0.007) revealed significant associations with disease severity. Chest computed tomography scans predominantly revealed inflammatory features (*n* = 81, 89.0%). Notably, patients classified as having mild or moderate conditions exhibited eventual improvement, while 13 patients (39.4%) among the severe cases succumbed to the disease.

**Conclusion:**

The classification of disease among patients aged 85 years or older old is correlated with advanced age, concurrent rheumatic immune diseases, and prolonged administration of endocrine medications. Furthermore, patients with elevated levels of LDH, IL-6, and D-dimer demonstrated a higher propensity for developing severe diseases.

## 1 Introduction

Since December 2019, a new strain of severe acute respiratory syndrome coronavirus 2 (SARS-CoV-2), referred to as COVID-19, has proliferated worldwide. As of September 13, 2023, globally 770 million confirmed cases of COVID-19 infection, with 6.95 million fatalities have been reported (1). By December 2022, China underwent a COVID-19 pandemic, with the prevailing strain being the Omicron variant (2).

Previous studies have indicated that clinical presentations of COVID-19 infection span from asymptomatic or mild symptoms to severe lung involvement, multi-organ dysfunction, and mortality (3). The Omicron variant has demonstrated significantly heightened immune evasion capabilities and transmissibility compared to earlier variants. However, its pathogenicity has diminished, with post-infection clinical manifestations shifting from predominant pneumonia to upper respiratory tract involvement (4–6). Correspondingly, the severity rate attributed to Omicron infection has notably decreased, owing to viral evolution, global vaccine dissemination, and enhanced vaccination rate (4). However, older adult patients continue to exhibit elevated severity and mortality rates. Xing et al. (7) conducted a study involving 181 hospitalized patients aged 80 years or older, revealing that 55.8% of them presented with severe or critical COVID-19 pneumonia. Numerous studies have underscored that older adult patients often present with multiple comorbidities such as hypertension and diabetes, necessitating prolonged medication use, including antihypertensive and hypoglycemic agents. Additionally, they frequently manifest weaknesses, compromised immune function, and diminished functional capacity, rendering them more susceptible to COVID-19 infection and its associated severity and mortality rates (8–10). In the early stages of the COVID-19 pandemic, it was reported that the mortality rate among patients aged 80 years or older was six times higher than that of younger patients (11). Based on projections from the 2022 World Population Outlook, the population aged 80 years or older is anticipated to constitute approximately 2% of the global population by 2021. Benefiting from advances in economic and medical infrastructure, this demographic is expected to grow over time (12).

As of now, most COVID-19 research has focused on younger and middle-aged populations, leaving a notable gap in clinical studies pertaining to patients aged 85 years or older. Consequently, there is no unanimous conclusion regarding the distinct clinical features, disease classification, and associated risk factors within this unique demographic affected by COVID-19. In light of the ongoing persistence and mutation of the virus, it is imperative to continue clinical inquiries into COVID-19 infection among the super-senior demographic, defined as age ≥ 85 years (13, 14). In the present study the clinical profiles of super-senior patients hospitalized due to varying presentations of COVID-19 are retrospectively examined and factors influencing disease classification are elucidated, providing valuable insights for clinical diagnosis and treatment.

## 2 Method

### 2.1 Study design and study object

**Figure 1:**
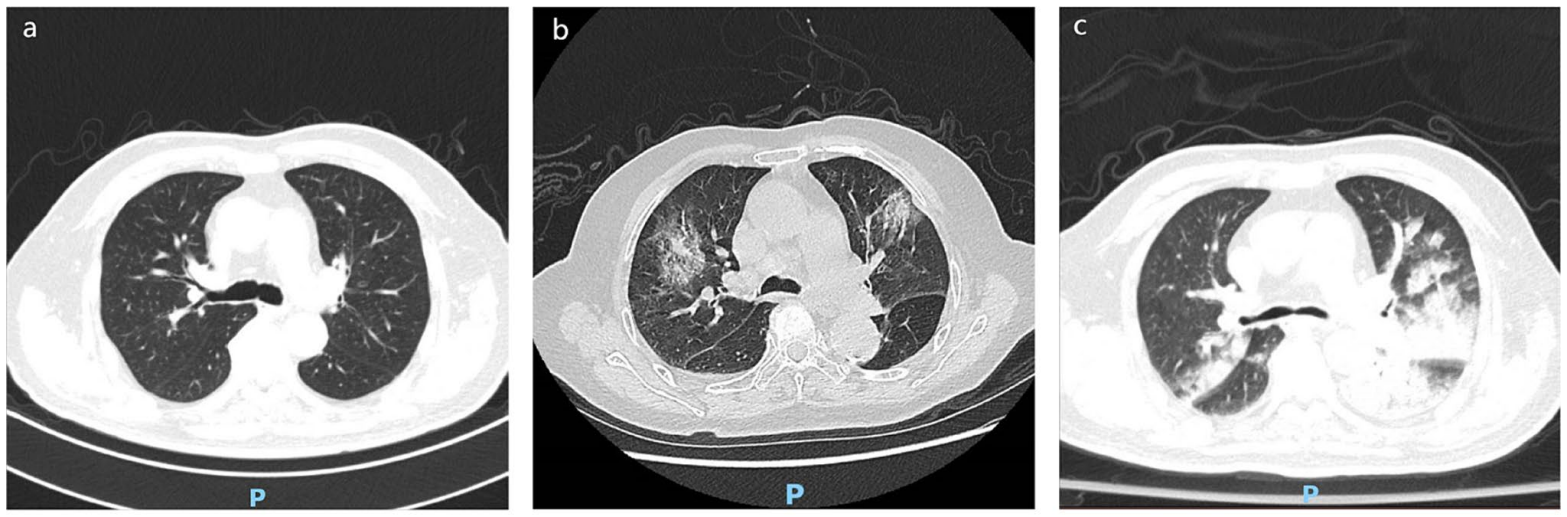
Different imaging findings of COVID-19 infection pneumonia. **(A)** Mild: absence of COVID-19 infection manifestation in imaging. **(B)** Moderate: multiple small spot shadows and interstitial changes in both lungs, which are always in the outer lung zone. **(C)** Severe: significant lesion progression (>50%) during the disease course, multiple ground glass shadows and infiltrating shadows in both lungs, lung consolidation and pleural effusion.

This retrospective study was conducted at Zhejiang Hospital, a general geriatric hospital in Hangzhou, China, with approval for the case series granted by the Hospital Institutional Ethics Committee of Zhejiang Province [2023 Clinical Review No. (46 K)]. The electronic medical records of hospitalized patients aged 85 years or older with COVID-19 during the period from December 20, 2022 to February 1, 2023, were retrospectively analyzed. In accordance with the COVID-19 Infection Diagnosis and Treatment Plan (10th edition for trial) of China, disease classification was based on clinical symptoms, signs, and imaging manifestations, encompassing mild, moderate, severe, and critical types. However, various clinical symptoms and signs in super-senior patients often manifest atypically due to organ decline. Furthermore, during the COVID-19 epidemic, many medical staff and workers were infected, resulting in a shortage of hospital personnel within a short timeframe, leading to simplification of some clinical information records. Consequently, for disease severity in this study, we primarily relied on imaging diagnosis. Diagnosis criteria for patients included a positive RT-PCR or antigen detection of SARS-CoV-2: absence of COVID-19 infection manifestation in imaging (mild), presence of characteristic COVID-19 infection manifestation in imaging (moderate), and presence of characteristic COVID-19 infection manifestation in pulmonary imaging with significant lesion progression (>50%) during the disease course (severe). Image findings are shown in Figures 1A–C. The manifestation of COVID-19 pneumonia chest computed tomography (CT) include: (1) multiple small spot shadows and interstitial changes in both lungs, which are always in the outer lung zone; (2) multiple ground glass shadows and infiltrating shadows in both lungs; (3) lung consolidation and pleural effusion. The diagnosis of pulmonary inflammation here was based on the definition in the Chinese Diagnosis and Treatment Protocol for Novel Coronavirus Infection (Trial Version 10): CT scans showed multiple small patchy shadows and interstitial changes in both lungs, with obvious peripheral distribution. This May progress to multiple ground-glass opacities and infiltrates in both lungs, with rare occurrence of pulmonary consolidation and minimal pleural effusion in severe cases. Exclusion criteria encompassed patients below 85 years old and those with serious underlying diseases, such as gastrointestinal bleeding, advanced malignant tumors, hemodialysis, or acute cardiovascular and cerebrovascular diseases prior to COVID-19 infection, and who were in an unstable condition, with the primary causes of poor prognosis unidentified.

### 2.2 Data collection

The electronic medical records of patients were reviewed, and relevant data were extracted, including basic demographic information such as gender and age. Given that super-senior patients commonly present with multi-system diseases and diverse diagnoses, meaningful data comparison would have been hindered if diseases were categorized individually. Therefore, our study team opted to classify diseases based on eight systems of the human body, including neurological diseases, circulatory diseases, respiratory diseases, endocrine diseases, motor diseases, rheumatic immune diseases, digestive diseases, and urogenital diseases. Additionally, concomitant medication was categorized based on different systems. However, apart from drugs related to the eight systems, medications pertaining to mental illness and nutritional supplements were separately listed. Furthermore, clinical symptoms at the onset, laboratory test results, and treatment plans of patients were reviewed, and their clinical outcomes were monitored. All data underwent verification by two researchers and were documented in a corresponding form. In cases of discrepancies, data were rechecked and confirmed.

### 2.3 Study progress

Based on the diagnostic criteria outlined in disease guidelines, nucleic acid samples from patients suspected with COVID-19 infection were obtained using oropharyngeal and/or nasopharyngeal swabs. A positive test result confirmed the diagnosis of COVID-19. In all cases reviewed in this study, patients tested positive for COVID-19 based on nucleic acid test results. Additional laboratory assessments included complete blood count, serum biochemical tests, inflammatory markers [including serum amyloid A, high sensitivity C-reactive protein, ferritin, and interleukin-6 (IL-6)], coagulation function, D-dimer levels, serum calcium ion concentration, and serum magnesium ion concentration, which were conducted either on the day of admission or the following day. It is important to note that among the patients reviewed, 59 were admitted to the hospital for testing on the same day or the following day of symptom onset, 16 patients underwent testing 2–7 days after symptom onset, and another 16 patients did not undergo testing until their first visit within 1–2 weeks after symptom onset. All patients underwent at least one chest CT scan during hospitalization, with those diagnosed with pulmonary COVID-19 infection undergoing two additional examinations. The radiological diagnoses of patients were independently verified by two experienced radiologists.

### 2.4 Data analysis

Continuous variables are presented as median and interquartile range (IQR), while categorical variables are expressed as absolute and relative frequencies. Descriptive univariate analysis was conducted for all variables. Pearson’s chi-squared test was used to compare independent samples for qualitative variables, with the Fisher’s exact probability method used when sample size criteria were not met. Quantitative variables were analyzed using the Mann–Whitney *U* test or Kruskal–Wallis test. Statistical analyses were carried out using SPSS 26.0 software, with a bilateral *p*-value less than 0.05 considered statistically significant.

## 3 Results

### 3.1 Demographic and clinical characteristics

A total of 91 patients, aged between 85 and 99 years, met the inclusion criteria for the study. The median age of the patients was 92 years (interquartile range 90–93), with 46 of them being male (50.5%). In this study, none of the patients have been vaccinated against COVID-19. Disease severity among the patients with COVID-19 was categorized based on imaging diagnostic criteria. The study population consisted of 10 mild cases (11%, 2 males), 48 moderate cases (52.7%, 25 males), and 33 severe cases (36.3%, 19 males). Their basic demographic and epidemiological characteristics are detailed in Table 1. Regarding demographic characteristics, age exhibited a significant association with the distribution of disease severity, with severe patients displaying a higher median age (mild: 89 years, moderate: 92 years, severe: 93 years, *p* = 0.011 < 0.05). However, gender had minimal impact on disease severity. Concerning concurrent underlying diseases, their diseases were categorized based on eight systems of the human body, with circulatory diseases (96.7%), urogenital diseases (72.5%), and neurological diseases (69.2%) being the most common complications observed in this population. Notably, the analysis revealed that comorbidity with rheumatic immune diseases significantly influenced disease severity distribution (mild: 80%, moderate: 39.6%, severe: 57.6%, *p* = 0.04). Furthermore, no significant impact of the number of concurrent disease types, number of long-term medication types, or eating styles on disease severity distribution was observed. An analysis of concomitant medication for each major system revealed a significant association between the use of endocrine drugs and disease severity distribution (mild: 0%, moderate: 43.8%, severe: 34.6%, *p* = 0.024). Further investigation revealed that hypoglycemic drugs were also significantly related to the distribution of disease severity (mild: 0%, moderate: 39.6%, severe: 27.3%, *p* = 0.034). A total of 59 patients (64.8%) were admitted to the hospital on the day of onset or the following day, while 16 patients (17.6%) were admitted 2–7 days after onset, and another 16 patients (17.6%) were admitted 1 week after onset. However, no significant correlation was observed between the time of presentation and the distribution of disease severity. The most common clinical symptoms reported were fever (87.9%) and cough (48.4%), followed by expectoration (19.8%), fatigue (16.5%), and poor appetite (8.8%).

**Table 1:** Demographic and epidemiological characteristics of patients aged 85 years and older with COVID-19 (*n* = 91).

Characteristics	Patients (*n* = 91)	Disease types			*p*-value
		Mild (*n* = 10)	Moderate (*n* = 48)	Severe (*n* = 33)	
Age (years old, median [IQR])	92(90–93)	89(88–90)	92(90–93)	93(91–96)	0.011*
Gender (male)	46(50.5%)	2(20%)	25(52.1%)	19(57.6%)	0.080
Complicating underlying diseases					
Nervous system	63(69.2%)	6(60%)	33(68.8%)	24(72.4%)	0.743
Circulatory system	88(96.7%)	10(100%)	46(95.8%)	32(97%)	1.000
Respiratory system	34(37.4%)	2(20%)	18(37.5%)	14(42.4%)	0.438
Endocrine system	47(51.6%)	2(20%)	25(52.1%)	20(60.6%)	0.079
Motor system	55(60.4%)	7(70%)	29(60.4%)	19(57.6%)	0.781
Rheumatic immune system	46(50.5%)	8(80%)	19(39.6%)	19(57.6%)	0.040*
Digestive system	64(70.3%)	7(70%)	32(66.7%)	25(75.8%)	0.679
Urogenital system	66(72.5%)	6(60%)	35(72.9%)	25(75.8%)	0.618
Disease types					
<3	9	1	5	3	0.289
3–4	22	2	13	7	
5–6	36	7	18	11	
>6	24	0	12	12	
Concomitant basic medicine					
Nervous system	39(42.9%)	4(40%)	20(41.7%)	15(45.5%)	0.927
Circulatory system	86(94.5%)	10(100%)	44(91.7%)	32(97%)	0.808
Respiratory system	6(6.6%)	0	3(6.3%)	3(9.1%)	0.846
Endocrine system	33(36.3%)	0	21(43.8%)	12(36.4%)	0.024*
Hypoglycemic drug	28(30.8%)	0	19(39.6%)	9(27.3%)	0.034*
Rheumatic immune system	32(35.2%)	5(50%)	15(31.3%)	12(36.4%)	0.520
Digestive system	58(63.7%)	6(60%)	29(60.4%)	23(69.7%)	0.671
Urogenital system	45(49.5%)	6(60%)	24(50%)	15(45.5%)	0.718
Psycho-related drugs	45(49.5%)	7(70%)	25(52.1%)	13(39.4%)	0.206
Drug type					
<3	16	0	9	7	0.414
3–4	30	6	16	8	
5–6	33	4	16	13	
>6	12	0	7	5	
Feeding way					
Oral	79	10(100%)	44(91.7%)	25(75.8%)	0.081
Nasal feeding tube	12	0	4(8.3%)	8(24.2)	
Course from onset to hospitalization					
<2 days	59(64.8%)	8(80%)	30(62.5%)	21(63.6%)	0.201
2–7 days	16(17.6%)	1(10%)	6(12.5%)	9(27.3%)	
>7 days	16(17.6%)	1(10%)	12(25%)	3(91.%)	
Onset symptoms					
Fever	80(87.9%)	9(90%)	41(85.4%)	30(90.9%)	0.808
Cough	44(48.4%)	4(40%)	25(52.1%)	15(45.5%)	0.720
Expectoration	18(19.8%)	2(20%)	9(18.8%)	7(21.2%)	0.963
Fatigue	15(16.5%)	2(20%)	8(16.7%)	5(15.2%)	0.935
Poor appetite	8(8.8%)	0	5(10.4%)	3(9.1%)	0.875

COVID-19, coronavirus disease 2019; IQR, interquartile range. *p*-values indicate differences between patients with mild, middle and severe cases.

### 3.2 Laboratory test results and radiological characteristics

All patients demonstrated improvement in their laboratory test results upon initial assessment, as detailed in Table 2. While all patients exhibited total white blood cell counts within the normal range, there were concurrent decreases in lymphocyte counts and increases in serum amyloid A (SAA); however, this did not significantly impact the distribution of disease severity. Notably, interleukin-6 (IL-6) levels were significantly elevated in moderate and severe patients (mild: 4.5 pg./mL, moderate: 22.2 pg./mL, severe: 20.7 pg./mL, *p* = 0.014), but no significant differences were observed in hypersensitive C-reactive protein (hs-CRP), procalcitonin (PCT), or ferritin concentrations between groups. Lactate dehydrogenase (LDH) levels were significantly elevated in severe patients (mild: 162 U/L, moderate: 193 U/L, severe: 212 U/L, *p* = 0.030), while no significant differences were noted in alanine transaminase (ALT), aspartate transaminase (AST), albumin levels, hemoglobin levels, or blood platelet counts between groups. Creatinine (Cr), glomerular filtration rate (eGFR), serum calcium ion concentration, and magnesium ion concentration were not significantly associated with disease severity distribution. Moreover, D-dimer levels were significantly higher in severe patients compared to the other two groups (mild: 0.19 mg/L, moderate: 0.23 mg/L, severe: 0.37 mg/L, *p* = 0.007), with no significant differences observed in serum fibrinogen concentration or prothrombin time. Additionally, all patients underwent at least one chest CT scan. Among them, 10 mild patients displayed no significant pulmonary imaging changes, while 48 moderate and 33 severe patients exhibited pulmonary inflammation. Furthermore, some severe patients presented with pleural effusion (26.4%) and pulmonary consolidation (14.3%), as indicated in Table 3. Notably, there was no significant difference in the distribution of pleural effusion among different disease severity, whereas pulmonary consolidation significantly increased in moderate and severe patients (mild: 0%, moderate: 8.3%, severe: 27.2%, *p* = 0.030).

**Table 2:** Laboratory test results of patients aged 85 years and older with COVID-19 (*n* = 91).

Characteristics	Patients (*n* = 91)	Disease types			*P*-value
		Mild (*n* = 10)	Moderate (*n* = 48)	Severe (*n* = 33)	
White blood cell count (×10^9^/L)	5.1(4.1–6.9)	4.25(3.5–5.5)	5.3(4.1–7.4)	5.7(4.1–6.8)	0.256
Lymphocyte count (×10^9^/L)	0.60(0.50–0.90)	0.65(0.60–0.78)	0.70(0.50–0.98)	0.60(0.50–0.90)	0.752
Hemoglobin (g/L)	113(104–125)	110(105–118)	112(103–123)	122(106–131)	0.288
Platelet count (×10^9^/L)	151(115–187)	161(136–210)	163(123–203)	137(105–164)	0.640
SAA (mg/L)	31.0(15.5–69.5)	28.5(17.0–38.9)	34.3(15.1–74.7)	31.6(17.6–109.4)	0.624
hs-CRP (mg/L)	9.72(3.87–19.68)	9.01(3.41–11.49)	7.37(3.59–18.31)	14.52(4.33–41.89)	0.338
PCT (ng/mL)	0.08(0.04–0.12)	0.06(0.02–0.13)	0.07(0.04–0.10)	0.08(0.05–0.19)	0.343
Ferritin (ng/ml)	204(98–435)	91(65–161)	206(95–399)	247(131–470)	0.992
IL-6 (pg/ml)	19.2(8.0–52.2)	4.5(4.0–11.2)	22.2(8.1–62.6)	20.7(10.3–50.9)	0.014*
Albumin (g/L)	34.3(32.1–37.4)	35.0(31.0–37.1)	34.2(32.1–37.4)	35.4(31.6–38.3)	0.881
ALT (U/L)	15.0(11.0–24.0)	12.5(10.8–17.0)	14.0(11.3–22.5)	17.0(11.5–30.5)	0.139
AST (U/L)	26(21–40)	26(22–27)	25(20–38)	35.5(22–49)	0.203
Cr (umol/L)	85(69–112)	74(62–99)	88(69–109)	84(68–126)	0.491
LDH (U/L)	199(164–225)	162(148–201)	193(160–223)	212(190–237)	0.030*
eGFR (ml/min × 1.73 m^2^)	61.9(44.3–73.5)	67.7(44.5–79.7)	62.6(44.5–72.3)	58.5(36.8–74.9)	0.667
Calcium ion (mmol/L)	2.17(2.07–2.28)	2.15(2.02–2.22)	2.17(2.07–2.30)	2.18(2.07–2.29)	0.623
Magnesium ion (mmol/L)	0.81(0.72–0.88)	0.74(0.71–0.90)	0.81(0.73–0.87)	0.81(0.73–0.88)	0.814
PT(s)	11.8(11.2–12.8)	11.7(11.0–12.6)	11.9(11.3–13.1)	11.9(11.0–12.4)	0.675
Fibrinogen (g/L)	3.53(3.00–4.11)	3.44(2.92–4.11)	3.51(2.98–4.10)	3.63(3.05–4.33)	0.804
D-dimer (mg/L)	0.27(0.17–0.43)	0.19(0.13–0.31)	0.23(0.16–0.38)	0.37(0.22–0.73)	0.007*

COVID-19, coronavirus disease 2019; IQR, interquartile range. *P*-values indicate differences between patients with mild, middle and severe cases.

**Table 3:** Imaging findings of patients aged 85 years and older diagnosed with COVID-19 (*n* = 91).

Characteristics	Patients (*n* = 91)	Disease types			*P*-value
		Mild (*n* = 10)	Moderate (*n* = 48)	Severe (*n* = 33)	
Imaging examination					
Inflammatory manifestation	81(93.1%)	0	48(100%)	33(100%)	0.000*
Manifestation of pleural effusion	24(26.4%)	0	16(33.3%)	8(24.2%)	0.086
Manifestation of consolidation	13(14.3%)	0	4(8.3%)	9(27.2%)	0.030*

COVID-19, coronavirus disease 2019; IQR, interquartile range. *P-*values indicate differences between patients with mild, middle, and severe cases.

### 3.3 Treatment plan and clinical outcome

Most patients received antiviral therapy (*n* = 66, 72.5%) and antibiotic therapy (*n* = 78, 85.7%). Symptomatic treatment varied, with 16 patients (17.6%) receiving non-steroidal anti-inflammatory drugs alone, 29 patients (31.9%) receiving glucocorticoid alone, and 31 patients (34.1%) receiving a combination of non-steroidal anti-inflammatory drugs and glucocorticoid. Significantly higher rates of antiviral therapy (mild: 40%, moderate: 71.8%, severe: 84.8%, *p* = 0.019) and antibiotic therapy (mild: 50%, moderate: 89.6%, severe: 90.9%, *p* = 0.012) were observed in severe patients compared to the other two groups. Moreover, the use of hormone drugs was significantly higher in severe patients compared to the other two groups, as depicted in Table 4. Ultimately, all 10 mild and 48 moderate patients exhibited improvement, while 13 out of 33 severe patients (39.4%) succumbed to the disease. Two patients died of sudden death; two gave up rescue because of respiratory failure; and the other nine were transferred to intensive care unit for treatment and finally died of multiple organ failure.

**Table 4:** Treatment and outcome of patients aged 85 years and older diagnosed with COVID-19 (*n* = 91).

Characteristics	Patients (*n* = 91)	Disease types			*P*-value
		Mild (*n* = 10)	Moderate (*n* = 48)	Severe (*n* = 33)	
Treatment (*n*[%])					
Antiviral therapy	66(72.5%)	4(40%)	34(71.8%)	28(84.8%)	0.019*
Antibiotic therapy	78(85.7%)	5(50%)	43(89.6%)	30(90.9%)	0.012*
Glucocorticoid	29(31.9%)	0	15(31.3%)	14(42.4%)	0.000*
Non-steroidal anti-inflammatory drugs	16(17.6%)	8(80%)	6(12.5%)	2(6.1%)	
Non-steroidal anti-inflammatory drugs and glucocorticoid	31(34.1%)	0	16(33.3%)	15(45.5%)	
With no non-steroidal anti-inflammatory drugs or glucocorticoid	15	2	11	2	
Outcome (*n*[%])					
Improvement	78(85.7%)	10(100%)	48(100%)	20(60.6%)	0.000*
Death	13(14.3%)	0	0	13(39.4%)	

COVID-19, coronavirus disease 2019; *P*-values indicate differences between patients with mild, middle, and severe cases.

## 4 Discussion

In this study, we delineated the clinical characteristics of COVID-19-infected patients aged 85 years and older across various disease severity, while also examining the associated risk factors influencing disease severity among super-senior patients. Four key findings emerged: (1) Differential treatment regimens were observed among hospitalized super-senior patients with COVID-19 based on disease severity, with significantly higher utilization rates of hormones, antiviral drugs, and antibiotics noted in severe cases compared to the other two groups. Furthermore, distinct patient outcomes were evident across different disease severity. (2) While the COVID-19 Infection Diagnosis and Treatment Plan (10th edition for trial) of China identifies age greater than 65 as a high-risk factor for severe diseases, our study highlights that even minor age differences within the super-senior population exert significant impacts on disease severity distribution. Advancing age correlates with heightened susceptibility to severe diseases. (3) Notably, among super-senior patients with multiple underlying diseases, common circulatory and neurological conditions demonstrated no significant influence on COVID-19 type distribution. However, rheumatic immune diseases emerged as potential factors impacting disease severity. (4) Consistent with previous findings, older adult patients exhibited lower-than-normal average lymphocyte levels, which did not significantly influence disease severity. Conversely, elevated levels of LDH, IL-6, and D-dimer were identified as potential risk factors for severe diseases.

The results of this study revealed that among super-senior patients with COVID-19 who were admitted to hospitals, treatment strategies varied depending on disease severity. Notably, the utilization rates of hormones, antiviral drugs, and antibiotics were significantly higher in severe cases compared to mild and moderate cases, with distinct outcomes observed among patients with different disease severity. Current studies, both in China and internationally, converge on a consensus regarding COVID-19 treatment protocols, encompassing seven key aspects: anti-inflammation, antiviral therapy, anticoagulation, management of acute hypoxic respiratory failure, administration of anti-SARS-CoV-2 (neutralizing) antibodies, regulation of the renin-angiotensin-aldosterone system, and supplementation of vitamins. Each treatment modality must be tailored to account for patient demographics and clinical manifestations (15). Regarding anti-inflammation, the COVID-19 Treatment Randomization Evaluation (RECOVERY) trial conducted the largest randomized controlled trial to date, demonstrating the efficacy of dexamethasone in reducing patient mortality rates (16). Additionally, after extensive antiviral drug research spanning over 2 years, Remdesivir (17), Nirmatrelvir/Ritonavir (18), and Molnupiravir (19) have been proven effective against COVID-19. Notably, older adult patients with COVID-19 frequently exhibit concurrent bacterial infections, necessitating the adjunctive use of antibiotics in clinical treatment. However, uncertainties persist regarding the optimal timing of antibiotic administration (20). This study underscores the variance in treatment plans among patients with different COVID-19 types, with significantly higher utilization rates of hormones, antiviral drugs, and antibiotics observed in severe cases compared to mild and moderate cases. However, despite intensive treatment, the prognosis for severe patients was less favorable than that for patients with milder conditions, likely attributable in part to the natural progression of the disease or, to some extent, inappropriate medication utilization. Previous studies have documented instances of improper use of Nirmatrelvir/Ritonavir during the Omicron strain epidemic in China (21).

Prior research has consistently highlighted age as an independent factor for the severity and mortality risk of COVID-19. However, many studies have predominantly focused on comparing outcomes across broad age categories, such as young adults, middle-aged, the older adult, and the very older adult. An extensive international study, spanning data from 52 countries from January 2020 to January 2022, revealed a robust correlation between age and the risk of death from COVID-19 on a global scale. This study revealed a pronounced escalation in the risk of death associated with advancing age, with every decade increment contributing to a 50% increase in the risk of mortality. Notably, patients over 90 years old exhibited a staggering 17-fold higher risk of death compared to those aged 20–30 (22). In a study conducted by Xing et al. (7), comprising 180 patients aged 80 years and older, it was observed that 55% of patients infected with COVID-19 developed severe or critical illness, with 35.9% succumbing to the infection within the study period. Barthel score, creatinine level, white blood cell count, D-dimer level, and glucocorticoid usage emerged as significant independent factors associated with the risk of mortality within 30 days (7). Contrary to previous findings, our study focusing on patients aged 85 years and older revealed distinct outcomes. Specifically, we observed that 52.7% of patients with COVID-19 exhibited moderate clinical manifestations, while 36.2% presented with severe clinical manifestations. Notably, the mortality rate among severe patients was markedly lower at 39.4% compared to reports from previous studies. Such disparities in findings May be attributed to variations in healthcare systems, ethnic characteristics, and geographical factors. Furthermore, our study underscored that within the special population aged 85 years and older, disease severity remained closely associated with age, with advanced age correlating with a heightened likelihood of experiencing severe diseases.

In the context of super-senior patients with COVID-19 and concurrent multiple underlying diseases, we discovered that prevalent cardiovascular and neurological conditions did not exert any discernible influence on the distribution of disease severity. However, rheumatic immune diseases emerged as a significant factor impacting disease severity. Since the onset of the COVID-19 pandemic, concerns have been raised regarding the potential relationship between rheumatic diseases and COVID-19 infection. Recent investigations have yielded generally consistent findings concerning the association between systemic rheumatism and adverse outcomes in patients with COVID-19. For instance, Siegel et al. (23) conducted a retrospective study involving 3,710 adult patients with SARS-CoV-2 infection in a New York Hospital. Their findings indicated a 24% increase in the likelihood of severe COVID-19 infection among patients with rheumatic immune diseases compared to the general population (23). Similarly, a nationwide group study conducted in Greece revealed that complications such as rheumatoid arthritis, systemic lupus erythematosus, and Sjogren syndrome were associated with a heightened risk of hospitalization due to COVID-19 infection, indicating a potentially more severe impact of COVID-19 on patients with systemic rheumatism when compared to the general population (24). Given the aberrant immune regulatory function inherent in rheumatic immune diseases and the potential influence of COVID-19 alongside the complexities introduced by hormone therapy and related immunosuppressants during treatment, a more protracted investigation May be warranted to comprehensively elucidate the post-infection status of patients with rheumatic immune diseases.

During the initial phase of the COVID-19 pandemic, hyperinflammation was recognized as a pivotal factor contributing to the severity of the disease (25). Previous investigations have established a correlation between elevated levels of neutrophils, C-reactive protein (26), serum LDH, IL-6 (27), and D-dimer in patients with COVID-19, alongside decreased lymphocyte levels, with disease severity and heightened mortality risk (28). In our study, we observed consistently low lymphocyte levels in older adult patients, albeit with no significant impact on the disease severity of super-senior patients with COVID-19 or the prognosis of severe cases. However, it is evident that lymphopenia constituted a characteristic feature of COVID-19 in older adult patients, potentially attributable to immune aging (29). Notably, our findings corroborate those of previous studies. Our investigation also revealed a significant association between elevated levels of LDH, IL-6, and D-dimer and an increased risk of severe diseases. However, these biomarkers did not exert a significant influence on the disease prognosis among severe patients.

Some studies have developed models to predict prognosis of COVID-19 patients by using clinical data and biomarkers, aiming to help clinicians identify high-risk patients. For example, FIB-4 is considered to be an effective predictor (30, 31). In our study, we tried to analyze the six factors, advanced age, concurrent rheumatic immune diseases, prolonged administration of endocrine medications, LDH, IL-6 and D-dimer. Due to the particularity of this super-senior demographic, the sample size we collected was limited. Therefore, it was difficult to establish a suitable model.

As a retrospective study, this investigation is subject to certain limitations. Firstly, the sample size of this study was relatively small, which May have compromised the robustness of our data analysis and limited our ability to draw definitive conclusions. Secondly, the study was conducted at a single institution, potentially limiting the generalizability of our findings to the broader population of patients aged 85 years and older with COVID-19. Lastly, certain important variables such as vaccination status, anticoagulation therapy, and nutritional status of patients were not included in our analysis, thereby potentially influencing the assessment of disease outcomes to some extent. These limitations should be acknowledged when interpreting the findings of this study.

## 5 Conclusion

The incidence of severe COVID-19 infection remains notably elevated among super-senior patients. Despite the limited sample size of this study, valuable insights can still be inferred. Current research indicates that disease severity among COVID-19 patients aged 85 years and older May be associated with advancing age, the presence of concurrent rheumatic immune diseases, and prolonged usage of hypoglycemic medications. Furthermore, individuals exhibiting elevated levels of LDH, IL-6, and D-dimer are at increased risk of developing severe diseases.

## Data Availability

The original contributions presented in the study are included in the article/supplementary material, further inquiries can be directed to the corresponding author.
